# An integrated genomic approach for the study of mandibular prognathism in the European seabass (*Dicentrarchus labrax*)

**DOI:** 10.1038/srep38673

**Published:** 2016-12-08

**Authors:** Massimiliano Babbucci, Serena Ferraresso, Marianna Pauletto, Rafaella Franch, Chiara Papetti, Tomaso Patarnello, Paolo Carnier, Luca Bargelloni

**Affiliations:** 1 Department of Comparative Biomedicine and Food Science, University of Padova, Viale dell’Università 16, 35020 Legnaro, Italy; 2 Department of Biology, University of Padova, Via Ugo Bassi 58/B, 35121 Padova, Italy

## Abstract

Skeletal anomalies in farmed fish are a relevant issue affecting animal welfare and health and causing significant economic losses. Here, a high-density genetic map of European seabass for QTL mapping of jaw deformity was constructed and a genome-wide association study (GWAS) was carried out on a total of 298 juveniles, 148 of which belonged to four full-sib families. Out of 298 fish, 107 were affected by mandibular prognathism (MP). Three significant QTLs and two candidate SNPs associated with MP were identified. The two GWAS candidate markers were located on ChrX and Chr17, both in close proximity with the peaks of the two most significant QTLs. Notably, the SNP marker on Chr17 was positioned within the *Sobp* gene coding region, which plays a pivotal role in craniofacial development. The analysis of differentially expressed genes in jaw-deformed animals highlighted the “nervous system development” as a crucial pathway in MP. In particular, *Zic2*, a key gene for craniofacial morphogenesis in model species, was significantly down-regulated in MP-affected animals. Gene expression data revealed also a significant down-regulation of *Sobp* in deformed larvae. Our analyses, integrating transcriptomic and GWA methods, provide evidence for putative mechanisms underlying seabass jaw deformity.

The European seabass (*Dicentrarchus labrax*) is one of the first non-salmonid species in marine aquaculture, with an annual production greater than 155,000 tons in 2014^1^. Despite great progress, several problems still limit the performance of sea bass farming, such as infectious diseases and larval and juvenile mortalities, often linked to anomalies in development. In the present paper, the focus is on a developmental malformation, mandibular prognathism (MP), in which the lower jaw is longer than the upper one providing a very distinctive underbite facial phenotype. In intensively reared European seabass, MP is a sporadic event and despite an incidence of 11% was reported in literature^2^, to our best knowledge seabass farms rarely experience incidences higher than 2%. In general, skeletal anomalies in farmed fish are a relevant problem entailing economic as well as animal health and welfare issues. For instance, in species that are mainly marketed as whole-fish, such as seabass, an anomalous external morphology could substantially impact the consumers’ overall perception of the product. Thus, fish need to be checked frequently and the deformed ones manually removed, even at large-scale production sites. Such defective fish are generally downgraded with consequent loss of profit.

Several external variables (*e.g.* nutritional unbalances, toxicants in water, mechanical stress, high water temperature) have been proposed as causative factors for skull and spinal malformations in cultured fish^3^,^4^,^5^. In European seabass, for example, it was shown that both temperature and different water-current speed had a significant effect on the incidence of lordosis, a severe vertebral deformity^6^. It is seemingly established that if a genetic base for skeletal anomalies is observed, this phenotype is expressed only when exceptional environmental conditions occur (i.e. intensive farming)^4^.

With regard to jaw anomalies, it has been suggested that they develop mainly during the early larval stages^3^,^4^,^7^. In farmed seabass, skull and skeletal malformations were shown to be associated with excess of vitamin A^8^ and with low phospholipids^8^,^9^ in the diet, although MP was not reported having higher frequency under such dietary conditions. Genetic factors also appear to be involved in fish skeletal deformities, as quantitative genetic studies in different species documented additive genetic variation underlying skeletal anomalies^10^ (and references therein). In the European seabass, the genetic basis of skeletal malformations was evaluated across different on-growing sites using the same genetic material^11^. Moderate heritability was observed for spine anomalies with varying incidence at different locations, although with negligible GxE interactions. Skull deformities were not reported and no data is available on genetic factors influencing jaw deformities in seabass. On the other hand, a genetic component for lower jaw anomalies has been proposed for other vertebrate species. In humans, prognathism typically shows familial aggregation and it is considered a multifactorial and polygenic trait likely explained by a threshold model^12^. Notably, it has been recently demonstrated that mutations at specific loci might be associated with MP. For instance, genes encoding matrilin 1 (MATN1) and fibroblast growth factor (FGF23) were proposed as candidate loci for MP^13^,^14^. In other mammalian species (dog, cattle, ass, horse) as well, lower jaw protrusion is considered a genetic disorder (Online Mendelian Inheritance in Animals database, http://omia.angis.org.au/home/), although only recently a genome-wide association study (GWAS) on prognathous horses has identified a candidate genomic region on equine chromosome 13 (ECA13)^15^.

The present study was prompted by the observation of two batches of seabass larvae showing high incidence of prognathism (>40%) at a commercial hatchery. As such batches experienced identical environmental conditions as several other batches of similar age that were minimally affected by the same malformation (<2%), a genetic predisposition in prognathous fish was suspected. In fact, each batch originated from fertilized eggs from single mass-spawning events, which are known to be dominated by a single dam and few sires^16^.

Samples for gene expression analysis of deformed and normal larvae from the affected batches were collected as soon as the malformation was evident (38 days post hatching, dph). The same sampling was repeated on early juveniles at 58 dph. Finally, at 79–83 dph individual fish were sampled, photographed, and fin-clips collected for genetic analysis. Here, we report on the results of the integrated analysis of prognathism in seabass based on transcriptome profiling and GWAS of the collected fish.

Despite the incidence of MP in farmed seabass is relatively low compared to other skeletal malformations in teleost fish^2^,^4^, this case-study represents an example of how an integrated genomic approach might identify the molecular mechanisms underlying developmental anomalies and provide genetic tools to potentially mitigate the damage.

## Results and Discussion

### Family-based genetic analysis

**Figure 1:**
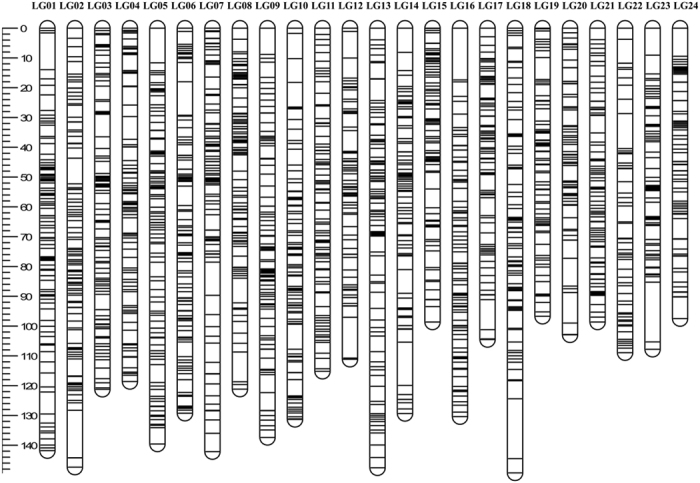
Genetic lengths and marker distribution of 24 linkage groups (LGs) in the sex-averaged linkage map of the European seabass.

Microsatellite-based parentage assignment identified four main medium-sized full-sib (FS) families originated from four sires and two dams (Table 1) and a large number of smaller ones. For the four FS families, 2bRAD sequencing was carried out also on parental DNAs, in order to construct a high-density linkage map and to search for QTLs associated with jaw deformity. The sequencing data obtained in our study was deposited in the NCBI-short read archive (SRA) database under the accession number SRP076258. A catalogue containing 7,390 loci was constructed with only data from parents, and used as reference for SNP discovery and genotyping to map families. A total of 5,304 SNP markers were identified and genotyped in more than 80% of the progeny. Genotyped SNPs were used to build a linkage map, after removing markers with distorted segregation. The number of informative SNPs in the mapping panel was 3,266. These markers were distributed over 24 Linkage Groups (LGs) in a sex-averaged linkage map, using a LOD = 9 as threshold for mapping data (Fig. 1 and Table 2). The total genetic length of the map was 2,787 cM. The genetic length of individual LGs ranged from 96 cM for LG19, containing 116 markers, to 147 cM for LG02, containing 171 markers, with an average of 116.12 cM. SNP markers of each LG were mapped against the genome of European seabass. As reported in Table 2, all LGs had a perfect match on a single chromosome of the seabass genome, except for LG21, which matched against two distinct chromosomes (Chr3 and Chr14). This discrepancy may be due to a not well-resolved LG21 or to a misassembly issue involving Chr03/Chr14. Notably, a total of 312 markers ranging from 3 (LG17) to 38 (LG6) mapped against ChrUN, which includes contigs not yet confidently placed on a specific chromosome.

Recently, a RAD-based linkage map using a mapping panel of 175 offspring that originated from a factorial cross between two dams and four sires from a single full-sib family was reported for the European seabass^17^, with 6,706 SNPs clustered in 24 LGs, and a total length of 4,816 cM. Both maps represent a substantial improvement over the previous linkage maps, which were based on microsatellite, AFLPs, and a limited number of SNP loci. Future integration of the two sets could help refine the genome assembly and provide ordered markers for genetic studies in seabass.

In the present study, the construction of a linkage map was instrumental to QTL mapping of jaw deformity. The maternal half-sib regression analysis identified a total of 18 QTLs significant either at genome-wide or chromosome-wide level (Table 3). Three QTLs, located on three different LGs, were genome-wide significant. The most significant one belongs to LG18 (P < 0.01), which corresponds to seabass Chr17 and explained 13.21% of total phenotypic variation. A second significant QTL was identified on LG22 (P < 0.05), matching ChrX, which explained 11.54% of total phenotypic variation. The third significant QTL was located on LG20 (P < 0.05), corresponding to Chr12 and accounting for 11.33% of the total phenotypic variation.

The same analysis, performed for trait “total length” (TL) highlighted eight significant QTLs (Table S1), four of them at 5% genome-wide level of significance. None of them overlapped with the MP-associated QTLs, thus putatively excluding, at genetic level, a correlation between both traits.

In addition, no significant coefficient of correlation was revealed between MP and TL at phenotypic level (r_pb_ = −0.0573, p-value = 0.33).

### GWAS for Mandibular Prognathism

**Figure 2:**
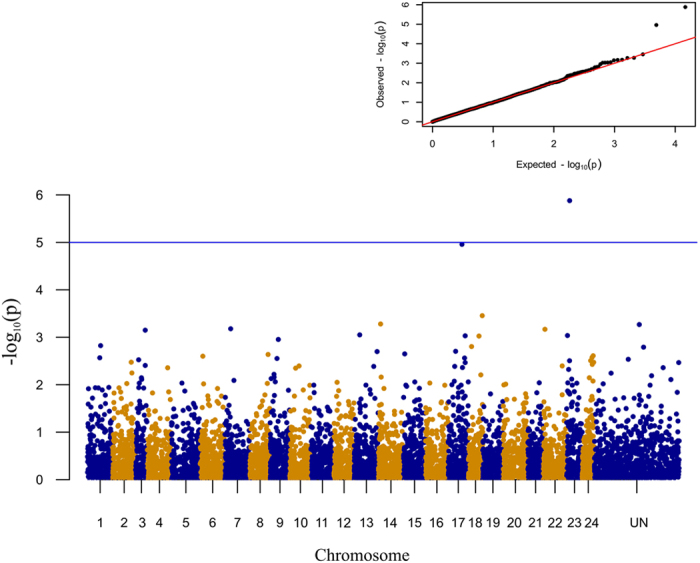
Manhattan plot for mandibular prognathism. A genome-wide case-control study showed a significant association of the phenotype prognathism on ChrX (labelled as Chr23) and a marginal significant association on Chr17. The blue line indicates the threshold level (−log10(1e-05)). The inset shows a quantile-quantile (qq) plot with the observed plotted against the expected p-values. The remaining unanchored scaffolds/contigs, those that could not be localized to a chromosome were concatenated into the virtual chromosome “UN” with 100 bp gaps between scaffolds.

A larger set of samples, including all the 298 juveniles collected from all families, was used for GWAS accounting for family structure. A total of 9,250 SNP markers was genotyped in more than 80% of the experimental population. After filtering for MAF < 0.05, 7,362 loci were retained for further analysis. All these SNPs mapped onto the seabass genome. The case-control allelic association analysis for MP was carried out with a mixed linear model and applied to 107 cases and 191 controls. Two SNPs, respectively on ChrX and Chr17, reached Bonferroni-corrected genome-wide significance level (P < 0.05) (Fig. 2, Table 4). These two candidate markers were in close proximity with the peaks of the two most significant QTLs on LG22 and LG18. GWAS results are based on a larger test panel than QTL analysis and include unrelated individuals. This suggests that the two identified regions could be involved in the determination of lower jaw deformity beyond a single family.

**Figure 3:**
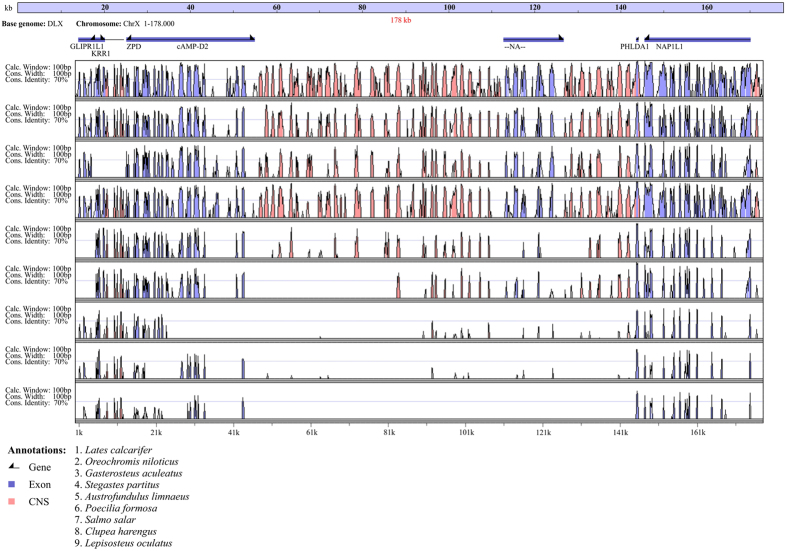
Comparison at different taxonomic levels of the ChrX genomic region flanking the significant locus L_39743.

The most significant association with prognathism was found for SNP marker L_39743 (P < 0.01 after Bonferroni correction), which was located at position 3,443,465 on ChrX within a putative gene showing an *in silico* predicted transcript (DLAgn_00206060) without any significant sequence similarity. The nearest genotyped SNP markers were positioned approximately 100 kilo base pairs (kbp) upstream (L_39738, ChrX:3,348,951) and downstream (L_39753, ChrX:3,540,808), defining a genomic region where four protein-coding genes are present (DLAgn_00206040, DLAgn_00206050, DLAgn_00206070, DLAgn_00206080). DLAgn_00206040 encodes a protein that contains a Fibronectin type 3 domain, a calcium-binding EGF-like domain, and a Zona pellucida-like domain and appears to be well-conserved within Acanthomorphs, but without a clearly identified ortholog in other vertebrates. DLAgn_00206050 codes for cAMP-regulated D2 protein-like (cAMP-D2), which has a putative orthologue in zebrafish (ZDB-GENE-060503–450, ENSDARG00000058492). Based on evidence from Ensembl Compara, ENSDARG00000058492 shows only one additional ortholog in cavefish. Paralogs originating from an ancestral gene duplication are present in several teleosts while no homolog is identified outside ray-finned fish. DLAgn_00206070 is the closest protein-coding gene to SNP L_39743 (<35 kbp downstream) and shows significant homology with *pleckstrin homology-like domain family a member 1* (*Phlda1*). In humans, *Phlda1* is associated with autosomal recessive intermediate osteoporosis where MP was reported in affected patients^18^. DLAgn_00206080 encodes a protein homologous to nucleosome assembly protein 1-like 1 (NAP1L1), which is involved in chromatin re-assembly. Remarkably, a genomic region of approximately 100 kbp without any known protein-coding gene spans between DLAgn_00206050 and DLAgn_00206070 and includes marker L_39743. Such a region, however, appears to be well conserved across several teleost genomes, with the presence of non-coding sequence elements showing high similarity especially within Acanthomorphs, the most derived teleost species (Fig. 3). Conserved non-coding elements (CNEs) have been found in comparisons of genomic regions at different taxonomic levels. Multiple lines of evidence suggest that CNEs have an important role in regulating gene expression, often encoding enhancers that act on nearby genes as well as distant ones and in several cases exerting their action on genetic loci involved in development^19^. It is therefore possible that the observed CNEs located between DLAgn_00206050 and DLAgn_00206070 are involved in transcriptional regulation. In turn, the genetic variant linked to L_39743 might contribute to lower jaw deformity altering patterns of gene expression rather than directly affecting the sequence of a protein coding gene. In fact, a recent study, using a combination of genomic technologies in mice, has shown the role of distant-acting enhancers in craniofacial development^20^. Although such evidence comes from a mammalian species, it is well recognized that despite the large evolutionary distance, the fundamental signaling pathways and cellular events that shape the craniofacial skeleton appear to be highly conserved from fish to human^21^.

The second significant marker (P < 0.05 after Bonferroni correction), L_12903, is positioned on Chr17 within the coding region of the gene *sine oculis-binding protein homolog* (*Sobp*). The closest upstream and downstream SNP markers were respectively L_51826 (Chr17:15,653,554) and L_12906 (Chr17:15,697,886) defining a relatively small region of approximately 45 kb, where four putative genes are located (DLAgn_00071270, DLAgn_00071280, DLAgn_00071290, DLAgn_00071300). DLAgn_00071270 encodes rho-associated protein kinase 2-like (ROCK2). ROCK2 is a protein kinase, which is a key regulator of actin cytoskeleton and cell polarity. The Rho kinase is involved in several biological processes including migration of neural cell precursors^22^ and survival of neural crest cells (NCC)^23^. NCC give rise to a great proportion of the tissues forming the vertebrate head and face. DLAgn_00071280 harbours, as already mentioned, marker L_12903 and codes for SOBP, a nuclear zinc finger protein whose molecular functions are only partially understood. Recessive mutations at the *Sobp* locus in mice cause defective patterning of sensory epithelium and deformed organ of Corti in the inner ear, while a homozygous missense mutation in *Sobp* has been associated with a syndrome causing mental retardation, anterior maxillary protrusion and strabismus^24^,^25^. SOBP may act as a critical transcription factor for neural development^26^. In fact, *Sobp* is included in the list of genes specifically expressed in NCC from the cranial mesenchyme, which play a key role in craniofacial development^27^. Two additional genes that characterize cranial mesenchyme NCC are located nearby marker L_12903, DLAgn_00071250 (Chr17:15,618,828–15,622,452) encoding leucine-rich alpha-2-glycoprotein, and DLAgn_00071210 (Chr17: 15,550,765–15,570,987) coding for apolipoprotein b-100.

Two additional genes are comprised between markers L_51826 and L_12906. DLAgn_00071290 seems to be expressed, but encodes a short protein sequence with a positive match of limited similarity with hypothetical proteins just in a few teleost species. DLAgn_00071300 encodes reticulon-4-interacting protein 1 mitochondrial-like (RTN4IP1), which has not been associated, until present, with any developmental process or malformation.

**Figure 4:**
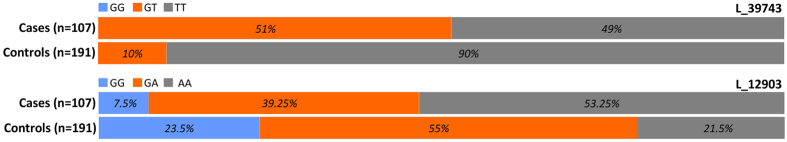
Genotype frequencies of the two best associated SNPs L_39743 and L_12903.

At locus L_39743, 55 (51%) prognathous fish were heterozygous for the minor allele and 52 (49%) were homozygous for the major allele. In control animals, 172 (90%) were homozygous for the major allele and 19 (10%) were heterozygous for the minor allele. At locus L_12903, 42 (39.25%) MP affected fish were heterozygous, 8 (7.5%) were homozygous for the minor allele while 57 (53.25%) were homozygous for the major allele. Among normal fish, 105 (55%) were heterozygotes, 45 (23.5%) were homozygotes for the minor allele, and 41 (21.5%) were homozygotes for the major allele (Fig. 4). The SNP effect calculated by GCTA was 0.23 for locus L_39743 and −0.16 for locus L_12903. A positive value means that the minor allele increases risk relative to phenotype, conversely a negative value represents a reduced risk (a protective effect of the minor allele). On the basis of the kinship matrix derived from the SNP data, the MP trait heritability was estimated using GCTA^28^. A moderate and significant value of 0.26 ± 0.08 (P-value = 9.6E-15) was found. Notably, this value is in agreement with estimates previously reported in the European seabass for skeletal deformities^11^.

A GWAS analysis carried out on growth trait pointed out two loci (not significant after Bonferroni correction) both located on European seabass Chr8 (Table S2). None of the significant GWAS loci associated to the MP were found on Chr8, excluding a putative correlation between both traits.

### Gene expression profiling of prognathous larvae and juveniles

Partial microarray data on gene expression profiles of the lower jaw at 58-days post hatching were already reported by^29^. Here, additional samples for the same developmental stage and novel data on larval heads at 38 dph are presented after the implementation of novel statistical tools to correct for array batch effects. Raw and normalized fluorescence data, including the newly added microarray experiments, were deposited in the GEO database under accession number GSE85056.

A two-class unpaired SAM analysis was performed to identify differentially expressed genes between normal and jaw deformed larvae (whole-head; 38 dph) and juveniles (dissected jaw; 58 dph). In the first case, 92 probes were significant at FDR < 0.05. Of these, 72 were more expressed in the whole head of prognathous larvae and 62 had a putative ortholog in zebrafish (Supplementary Table S3). In the case of dissected lower jaw, 708 probes, corresponding to 429 unique transcripts, were found significant, 21 (18 unique transcripts) being up-regulated and 687 (411 unique transcripts) being down-regulated in jaw deformed juveniles (Supplementary Table S4). The larger number of differentially expressed genes (DEGs) in the latter case suggests that dissecting the affected anatomical region substantially increased the discriminatory power of gene expression profiling. It might also be possible that, as fish grow, the defect becomes more pronounced, not only macroscopically, as already observed, but also in terms of affected genes/gene pathways.

To obtain a more comprehensive interpretation of the set of genes differentially expressed, a functional enrichment analysis was performed using the tool DAVID (see Methods). Six GO Biological Process (GO_BP) terms were significantly enriched, with marginal statistical support (Supplementary Table S5). The most numerous term was “metabolic process” with 26 genes. Of these, quite relevant was the putative ortholog of zebrafish ENSDARG00000074481, which encodes unc-51 like autophagy activating kinase 1 (ULK1b). ULK1 belongs to a kinase triad (AMPK, mTORC1, ULK1) that controls cell growth depending on energy and nutrient status^30^. More specifically, ULK1 is a key player in inducing autophagy, a process that is known to be essential for vertebrate development^31^,^32^.

A total of 172 putative zebrafish orthologs matched DEGs in the juvenile lower jaws. Using zebrafish functional annotations as a proxy for seabass, 38 GO_BP terms were found to be significantly enriched (Supplementary Table S5). Several terms involved microtubule-related processes and contained, in particular, four different genes encoding stathmin-like proteins (STMN1B, STMN2A, STMN2B, STMN4), which were all significantly down-regulated in deformed lower jaw. Stathmins are tubulin-interacting proteins involved in several cell functions^33^.

A potential link might also exist between stathmins and one of the genetic loci (DLAgn_00071270 encoding ROCK2) located in the candidate region of chromosome 17, since STMN and ROCK were shown to interact to control cell migration^34^. Stathmin mediates neuroblastoma metastasis in a tubulin-independent manner via RhoA/ROCK signaling and enhanced transendothelial migration^34^. Stathmins were also reported to be essential for neural cell differentiation^35^. The microtubule destabilizing protein stathmin controls the transition from dividing neuronal precursors to postmitotic neurons during adult hippocampal neurogenesis^35^. In fact, stathmins are also found in another significantly enriched GO_BP term, “nervous system development” (Supplementary Table S5), further confirming the role of neural cells in the development of jaw deformity. In total, 18 DEGs are included in this GO term. Of these, the most relevant gene is possibly DLAgn_00053460, the putative ortholog of zebrafish *Zic2a*. ZIC2 is a zinc-finger transcription factor, which is a key player in craniofacial development. In humans it is strongly linked to holoprosencephaly, the most common cranial malformation. In zebrafish, it was shown to play a dual role during craniofacial development contributing to two different aspects of craniofacial morphogenesis: i) neural crest induction and migration, and ii) early patterning of tissues adjacent to craniofacial chondrogenic condensations^36^.

Significant differential expression of a putative Z*ic2* ortholog in the deformed jaw provides evidence that altered functioning of NCC likely drives MP in seabass.

### Integrating gene expression profiles and genetic data

To further explore the potential links between transcriptomic characterization of jaw deformity and GWAS for MP, a dedicated analysis was performed to assess whether there was any perturbation of expression for genes located in the genomic regions nearby the two GWAS-significant genetic markers. Using a conservative approach, a relatively large target region was considered and a two-class SAM significant test was carried out on all probes mapping in the regions spanning ±500 kb respectively around loci L_39743 and L_12903 (Supplementary Table S6). At variance with evidence on a whole-transcriptome scale, fewer significant transcripts were observed in gene expression profiling of the lower jaw at 58 days than in whole-heads (38-days larvae), with three genes within region “L_39743” and one gene for region “L_12903”. Of these, two distinct probes indicated significant down-regulation of DLAgn_00206050, one of the four genes in the narrow region on ChrX between markers L_39738 and L_39753, encoding cAMP-D2, immediately upstream several CNEs (Fig. 3). Transcriptome profiling of whole-heads identified a much larger set of DEGs, with five genes located on the ChrX region and eight on the Chr17 one. One gene (DLAgn_00206010) showed substantial up-regulation in deformed larvae with fold-change > 6. DLAgn_00206010 codes for CMP-Neu5Ac hydroxylase (CMAH), an enzyme involved in the synthesis of sialic acid. CMAH enzyme activity was lost in humans, although *Cmah* expression was reported as (mesenchimal) stem cell marker^37^. In lower vertebrates (rainbow trout and *Xenopus laevis*) *Cmah* was found to be expressed in the ovary^38^, while *Cmah*-null mice showed hearing loss and several other abnormalities^39^.

Among the several DEGs in whole-heads located in the target region on Chr17, *Sobp* was found to be significantly down-regulated in deformed larvae, with concordant evidence from two probes (Supplementary Table S6). Such evidence further supports *Sobp* as a candidate gene contributing to mandibular prognathism.

In conclusion, in the present study, integration of transcriptomic analysis with GWAS provided evidence for the potential mechanisms underlying jaw deformity in the European seabass. QTL mapping and GWA analysis allowed the identification of two regions implied in the determination of lower jaw deformity and pointed out a candidate gene, *Sobp*, likely contributing to seabass MP. Moreover, the presence of a cluster of CNEs around to the most significant SNP on ChrX is suggestive since such elements might act as distant enhancers in craniofacial development.

Finally, as previously reported in model species, differential regulation of several genes involved in neural development, such as putative *Zic2a* and stathmins, confirms the importance of this biological process to develop craniofacial deformities.

The present work might be considered as a case-study proving the feasibility of an integrated genomic approach as a compelling strategy to unravel the molecular bases of skeletal anomalies in fish aquaculture. As a future perspective, these integrated methods could be pivotal to the development of genetic tools intended to be applied in breeding selection.

## Methods

### Ethics statement

No specific permits were required for the work described here. Individuals included in the present study were bought from a commercial hatchery and they were not subjected to any experimental manipulation. The study was performed in accordance with the EU directive 2010/63/EU and Italian DL 2014/26. The experiments, as well as the euthanasia procedure, were monitored and carried out by authorized staff to minimise animals’ suffering.

### Samples collection, phenotypes description and parental assignment

A total of 298 juveniles (79–83 days old, average standard length 4 cm) and 48 broodstocks were collected and analysed. All samples were provided by the fish farm “Cà Zuliani” (Pila di Porto Tolle, Italy). Jaw deformed phenotype was assigned to individual juvenile affected fish by two operators independently. Each fish was also photographed, weighed, and its length measured. Out of 298 seabass juveniles, 107 were affected by MP (191 unaffected). For all subsequent analyses (i.e. QTL and GWAS) the presence/absence of prognathism was coded as 1/0, respectively. Microsatellites analysis was performed on both adults and juveniles by using a set of 9 loci according to^40^ (see Supplementary Table S7). Briefly, alleles scoring was achieved by means of Genotyper v3.7 (Applied Biosystems) and the parental assignment test was assessed with the software Cervus (http://fieldgenetics.com) using default settings.

### 2b-RAD libraries preparation and sequencing

Genomic DNA (gDNA) was extracted from approximately 20 mg of tissue (fin clip) using the commercial kit Invisorb^®^ Spin Tissue Mini Kit (Invitek, STRATEC Biomedical, Germany) following the manufacturer’s recommendations. Genomic DNA concentration and purity were quantified by using both a NanoDrop ND-1000 spectrophotometer (Thermo Fisher Scientific, Waltham, Massachusetts, USA) and a Qubit 2.0 Fluorimeter (Invitrogen, ThermoFisher Scientific, MA, USA). This procedure ensured comparable concentrations of high quality gDNA as required for 2b-RAD library preparation.

A 2b-RAD library was constructed for each individual following a modification of the protocol from^41^. To assess the robustness of the method, two libraries were replicated (Technical replicates, TRs) for two individuals^42^. A total of 200 ng of gDNA from each sample were digested with 2 U *CspC*I (New England Biolabs, NEB, Ipswich, Massachusetts, USA) for 1 h at 37 °C. Sample-specific barcodes were designed with Barcode generator (http://comailab.genomecenter.ucdavies.edu) and introduced by PCR with platform-specific barcode-bearing primers. The concentration of each purified individual library was quantified using Qubit^®^ds DNA BR Assay Kit (Invitrogen, ThermoFisher Scientific, MA, USA) and Mx3000 P qPCR instrument, while the libraries quality was checked on an Agilent 2100 Bioanalyzer (Agilent Technologies, Santa Clara, California, USA). Individual libraries were pooled into equimolar amounts. Pooled libraries were sequenced on an Illumina HiSeq2500 platform with a 50 bp single-read module at the Genomix4Life S.r.l. facility (Baronissi, Salerno, Italy), which also performed data demultiplexing.

### SNP discovery and genotyping

Demultiplexed reads returned by the sequencing facility were quality-checked with the software FastQC (http://bioinformatics.babraham.ac.uk). Adapter trimming was performed by running a customized script (available upon request), thus obtaining 32-bp fragments ready to be evaluated for SNPs presence in STACKS. Filtered reads were then mapped against the European seabass genome^43^ with CLC Genomics Workbench version 8.5 (http://qiagenbioinformatics.com) mapping module. The following parameters were applied: *length fraction* = 1.0 and *similarity fraction* = 0.9 (all remaining parameters as default), retaining only uniquely mapped reads. Mapping results were exported in SAM format and used as input for *refmap*_*map.pl* in STACKS v. 1.36, a program for SNP discovery and genotyping^44^,^45^. Different settings were tested on the TRs dataset to fine tune the STACKS pipeline parameters and to assess the consistency of the results as in ref. 42. To construct stacks and catalogue loci, a minimum coverage of 10X was used for parental samples^45^. A maximum of two mismatches between stacks were allowed for catalogue construction. For the offspring, stacks were assembled with a minimum coverage of 5X sequencing reads. One SNP per locus and maximum 2 alleles allowed were chosen as parameters for the genotype data calling. Loci with more than 30% of missing data and with more than one SNP/locus were excluded from further analyses.

### Linkage map construction

An sex-averaged. linkage map was constructed using Lep-MAP2^46^, a software suite handling thousands of markers and multiple families. All the four full-sib families were used as input, and SNPs showing significant segregation distortion with P-value < 0.01 and a minor allele frequency MAF < 0.05 were excluded. The remaining markers were used for the linkage group assignment with LOD score thresholds of 6–12. Singular markers were then added to the LGs found using the *JoinSingles* module with a LOD score limit of 6. The order of markers was calculated with *OrderMarkers* module, with a recombination rate parameter of 0.40 and “useKosambi = 1” and “maxDistance = 50” options. The ordering task was performed 6 times and the order with the best likelihood was chosen following the author guidelines of LepMAP2 (https://sourceforge.net/p/lepmap2/wiki/Home/). The map was drawn using MapChart v 2.3^47^.

### QTL mapping for MP

The analysis was carried out using a half-sib regression on the MHS family, implemented in the online tool GridQTL (http://gridqtl.org.uk) and described by^48^. This software implements a multi-marker approach of interval mapping in half-sib families; notably, this method of QTL analysis does not assume the parents had fixed QTL alleles therefore relaxing this parameter^48^. The *F* statistic is given for each LG for the most likely position as well for the chromosome-wide and the genome-wide thresholds used to determine the significance of the detected QTL. Each threshold was obtained by 1000 permutations for the trait. A 95% confidence interval for each significant QTL (CI95) was determined using 10,000 bootstraps with resampling. A genome scan for QTLs for growth trait was also performed with GridQTL, using the same parameters described above. The estimated proportion of the phenotypic variance explained by each QTL (i.e., the estimated heritability due to the QTL) was calculated with the formula 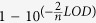, where *n* is the sample size^49^.

### Genome wide association study

The entire dataset of 298 juvenile seabass was used for GWA analysis. The case/control (107 cases and 191 controls) GWAS for MP was performed using a mixed linear model (suitable for binary traits as prognathism) implemented in GCTA^28^ and considering the relatedness of juveniles. The advantages of mixed-linear-model association (MLMA) method include the prevention of false positive associations due to population or relatedness structure and an increase in power obtained through the application of a correction that is specific to this structure^50^. Briefly, first three files (.bed, .bim and .fam) were generated for the GWAS genotypes using PLINK^51^; then a *–make-grm* option was used to generate grm.gz and grm.id files; a phenotype file for prognathism trait was prepared and the *–mlma* option was used to perform the MLMA association analysis. We considered genome-wide significance where P-values were below the 5% corrected threshold for 7,362 independent test. The adjusted P-values were determined with the function *p.adjusted* implemented in R-CRAN (https://cran.r-project.org). Variance explained by all SNPs was estimated with the software GCTA with a user-specified disease prevalence = 0.1^28^. First, a genetic relationship matrix (GRM) between pairs of individuals was calculated via the -*make-grm* function in GCTA, then a restricted maximum likelihood (REML) analysis was performed to estimate the phenotypic variance explained by the SNPs^52^,^53^. A Manhattan and Q-Q plot for GWAS data were created with the R-CRAN package *qqman* v. 0.1.2^54^.

Similarly, GWAS analysis for growth trait was carried out using GCTA with the same parameters as described above.

Conserved non-coding elements (CNEs) were investigated through mVISTA, a tool for comparative genomics analysis available online (http://genome.lbl.gov/vista/index.shtml). A point biserial correlation coefficient (r_pb_) was calculated to test the association, at phenotypic level, between prognathism and growth using *ltm* v. 1.0 package^55^. The correlation coefficient r_pb_ calculated is a measure of the strength of association between a continuous-level variable and a binary data.

### Microarray Gene Expression Analysis

Two seabass developmental stages: i) larvae (38 days-old, average length 12 mm), and ii) juveniles (58 days-old, average standard length 16 mm) were collected at the fish farm “Ca’ Zuliani” (Pila di Porto Tolle, Italy) and sacrificed using an excess of anesthetic, as previously described. For 38 dph larvae, the cranial regions were dissected under a stereomicroscope from15 normal and 15 jaw-deformed individuals and pooled (5 heads/pool) to obtain 3 independent pools per condition. For 58 dph juveniles, the lower jaws were dissected and pooled (5 jaws/pool) from a total of 50 individuals, 25 normal and 25 affected by jaw-deformity, thus providing 5 independent pools per condition. Gene expression experiments were performed using a single dye (Cy3) labelling scheme on the Agilent-019810 *D. labrax* oligo microarray (GEO accession: GPL9663) containing 19,035 unique transcripts^29^, each represented by two non-overlapping probes. Normalization procedures were performed using R statistical software. Microarray data were cyclic lowess (CL) normalized across all arrays and CL-normalized data were further adjusted for the known between-experiments batch effects by implementing parametric Combat correction in R^56^. A two-class comparison (FDR < 5%, fold-change ≥ 2) was carried out on Significance Analysis of Microarrays (SAM) software in order to identify differentially expressed genes between normal and jaw-deformed groups^57^. A functional interpretation of DEGs was obtained through enrichment analysis using the Database for Annotation, Visualization, and Integrated Discovery (DAVID) software version 6.8 beta^58^ with default parameters. Since DAVID database contains functional annotation data for a limited number of species, it was necessary to use the zebrafish feature as identifiers. To retrieve zebrafish IDs, a BLASTX search (cut off e-value < 1.0 e-5) was carried out against the high quality *D. rerio* draft genome stored on ENSEMBL database by using all seabass transcripts as query. These identifiers were used to define a “gene list” and a “background”, corresponding to the list of differentially transcribed seabass genes and to all the transcripts that were represented on the array, respectively.

## Additional Information

**Accession codes**: Sequencing data was deposited in the NCBI-short read archive (SRA) database under the accession number SRP076258. Gene expression data are available on Gene Expression Omnibus (GEO) repository under the accession number GSE85056.

**How to cite this article**: Babbucci, M. *et al*. An integrated genomic approach for the study of mandibular prognathism in the European seabass (*Dicentrarchus labrax*). *Sci. Rep.* **6**, 38673; doi: 10.1038/srep38673 (2016).

**Publisher's note:** Springer Nature remains neutral with regard to jurisdictional claims in published maps and institutional affiliations.

## Supplementary Material

Supplementary Information

Supplementary Dataset S3

Supplementary Dataset S4

Supplementary Dataset S5

Supplementary Dataset S6

## Figures and Tables

**Table 1:** Family structure with number of offspring per half-sib family and per full-sib family.

Sire	Dam	
	R41	R26
R45	97	—
R25	34	—
R12	17	—
R17	—	24
Total	148	24

**Table 2:** Summary statistics of the sex-averaged genetic map of European seabass.

LG	Sex-averaged map			
	Mapped Markers	Genetic length (cM)	Marker interval (cM)	Physical position
1	171	141	0.83	Chr16
2	171	147	0.86	Chr13
3	158	121	1.32	Chr05
4	148	118	0.80	Chr20
5	157	139	0.89	Chr06
6	162	129	0.80	Chr1B
7	147	142	0.97	Chr1A
8	137	121	0.89	Chr04
9	147	137	0.94	Chr02
10	145	131	0.91	Chr08
11	138	115	0.84	Chr10
12	131	111	0.85	Chr07
13	147	147	1.01	Chr15
14	140	129	0.93	Chr14
15	128	98	0.77	Chr (22–25)
16	134	130	0.98	Chr09
17	125	104	0.84	Chr11
18	130	149	1.15	Chr17
19	116	96	0.84	Chr19
20	108	102	0.96	Chr12
21	126	98	1.60	Chr03/Chr14
22	101	108	1.08	ChrX
23	107	107	1.01	Chr (18–21)
24	92	97	1.07	Chr24

Physical position of the linkage groups is referred to the European seabass genome.

**Table 3:** Summary statistics of the significant QTL for prognathism in European seabass.

QTL	LG	Position (cM)	Chr	F	Expl. Variation
MHS-01****	18	84	17	23.30	13.21%
MHS-02***	20	44	12	19.57	11.33%
MHS-03***	22	74	X	19.96	11.54%
MHS-04**	24	23	24	16.68	3.24%
MHS-05**	19	58	19	16.60	6.65%
MHS-06**	14	71	14	15.94	3.60%
MHS-07**	8	38	4	15.25	3.54%
MHS-08**	16	6	9	15.22	5.61%
MHS-09**	15	32	(22–25)	14.56	3.48%
MHS-10*	6	106	1B	14.37	2.47%
MHS-11**	17	12	11	13.36	3.38%
MHS-12**	5	79	6	12.90	3.33%
MHS-13*	4	111	20	11.71	2.21%
MHS-14*	23	22	(18–21)	11.07	3.14%
MHS-15*	21	28	03	11.05	3.14%
MHS-16*	7	71	1A	11.20	1.15%
MHS-17*	9	135	2	10.66	3.10%
MHS-18*	11	62	10	10.64	3.09%

MHS = maternal half-sib, LG = Linkage Group, cM = centimorgan, Chr = Chromosome, F = F-statistic.

^****^Genome-wide significant QTL (P < 0.01).

^***^Genome-wide significant QTL (P < 0.05).

^**^Chromosome-wide significant QTL (P < 0.01).

^*^Chromosome-wide significant QTL (P < 0.05).

**Table 4:** SNPs associated with mandibular prognathism using a case/control mixed linear model based association analysis.

SNP	Seabass chromosome	Position (bp)	Minor allele frequency	Harbouring gene	Nearest gene	p-value
L_39743	ChrX	3,443,463	G(0.11)/T	-NA-	PHLDA1	1.32E-6*
L_12903	Chr17	1,566,309	G(0.42)/A	SOBP	ROCK2	1.1E-5*

Significance after Bonferroni correction was highlighted by an asterisk. NA = not annotated.
