# Supplementary material for: An integrated genomic approach for the study of mandibular prognathism in the European seabass (Dicentrarchus labrax)
Source: Sci Rep. 2016 Dec 8;6:38673. doi: 10.1038/srep38673 (PMC5144136; doi:10.1038/srep38673)
Supplement: Supplementary Information [file srep38673-s1.doc]

**An integrated genomic approach for the study of mandibular prognathism in the European seabass (*Dicentrarchus labrax*).**

**Massimiliano Babbucci^1*^, Serena Ferraresso^1^, Marianna Pauletto^1^, Rafaella Franch^1^, Chiara Papetti^2^, Tomaso Patarnello^1^, Paolo Carnier^1^, Luca Bargelloni^1^.**

| **QTL** | **LG** | **Position (cM)** | **Chr** | **F** | **Expl. Variation** |
| --- | --- | --- | --- | --- | --- |
| MHS-01^***^ | 5 | 123 | 6 | 21.37 | 3,95% |
| MHS-02^***^ | 19 | 7 | 19 | 21.12 | 3.94% |
| MHS-03^***^ | 9 | 72 | 2 | 20.43 | 3.90% |
| MHS-04^***^ | 23 | 86 | (18-21) | 19.81 | 3.86% |
| MHS-05^**^ | 10 | 87 | 8 | 16.17 | 3.61% |
| MHS-06^**^ | 1 | 89 | 16 | 14.13 | 3.45% |
| MHS-07^**^ | 2 | 118 | 13 | 12.39 | 3.34% |
| MHS-08^*^ | 24 | 83 | 24 | 9.26 | 2.91% |

**Supplementary Table S1:** Summary statistics of the significant QTL for growth in European seabass. MHS= maternal half-sib, LG = Linkage Group, cM = centimorgan, Chr = Chromosome, F= F-statistic.

****Genome-wide significant QTL (P<0.05)*

***Chromosome-wide significant QTL (P<0.01)*

**Chromosome-wide significant QTL (P<0.05)*

| **SNP** | **Seabass chromosome** | **Position (bp)** | **Minor allele frequency** | **Harbouring gene** | **Nearest gene** | **p-value** |
| --- | --- | --- | --- | --- | --- | --- |
| L_37058 | Chr8 | 5,769,773 | G(0.13)/A | -NA- | WFIKKN2 | 2.5E-4*^ns^* |
| L_37059 | Chr8 | 5,775,389 | G(0.22)/A | TOB1 | WFIKKN2 | 3.2E-4*^ns^* |

**Supplementary Table S2:** SNPs associated with growth using a mixed linear model based association analysis. NA= not annotated.

*^ns^ = not significant after Bonferroni correction.*

*Excel file Supplementary Table S3*

**Supplementary Table S3:** Differentially expressed genes between normal and jaw deformed larvae (whole-head; 38 dph).

*Excel file Supplementary Table S4*

**Supplementary Table S4:** Differentially expressed genes between normal and jaw deformed juveniles (dissected mandible; 58 dph).

*Excel file Supplementary Table S5*

**Supplementary Table S5:** Functional enrichment analysis carried out on DEGs.

*Excel file Supplementary Table S6*

**Supplementary Table S6:** SAM significant test on all probes mapping in the regions spanning ± 500kb respectively around the two most significant GWAS analysis loci.

| **Locus** | **Repeat** | **Primers** | **T_a_** | **Size range** | **Alleles** |
| --- | --- | --- | --- | --- | --- |
| **DLA0008** | (AC)_24_ | F:AAGCTATCTGATCTCGCTTG  R:ACGTGATTAAGTGTTTGTGAG | 56 | 236-298 | 11 |
| **DLA0119** | (TG)_10_ | F:GCAGGTTCAAATTATTTTTGCTC  R:TCCTCCTTTTGCTTGCTAGG | 54 | 219-261 | 10 |
| **DLA0016** | (TG)_24_ | F:GTGACCGCAGATGAAGAAC  R:ACTGTGGGCTCATAAACATC | 54 | 228-258 | 11 |
| **DLA0020** | (TG)_20_ | F:GTCTAATGAGCAGTGGAGCAG  R:GCATGTTAGATCCACCTCTTTC | 56 | 153-175 | 8 |
| **DLA0105** | (AC)_16_ | F:GAGGCTGTATGCTGTTGCAG  R:ACCCATGCATAAGGTCAGTG | 56 | 138-172 | 9 |
| **DLA0145** | (TC)_20_ | F:CCCACAATAGATTCAAATAG  R:CACACATGCAATTATACTG | 54 | 152-188 | 10 |
| **DLA0248** | (TC)_5_ACAT(TC)_5_(T)_2_(TC)_7_  (AC)_3_(ACGC)_4_ | F:TGCATGATGATGTGTGAGCA  R:TGGCAGGCTAAAACCTCAAG | 54 | 111-127 | 5 |
| **DLA0228** | (AAAG)_3_(AG)_4_(AAAG)_3_ | F:CCAATGTTTTCATCCCCTCA  R:TTGCTGCTTGTGAAGTGACC | 54 | 86-98 | 3 |
| **DLA0244** | (TG)_12_(AG)_5_(TG)_2_ | F:ACTGAAAGCACAGCCTGGTT  R:CCCCCATCCAATACACTCAC | 54 | 100-104 | 3 |

**Supplementary Table S7: Microsatellites loci for parental assignment**. Locus name (Locus), repeats number (Repeat), primers sequence (Primers), annealing temperature (Ta), fragment length (Size range), alleles number (Alleles).
